# Rethinking Gleason 6 Prostate Cancer: Embracing PrNLMP for Precision and Progress

**DOI:** 10.1007/s11934-025-01285-1

**Published:** 2025-07-14

**Authors:** Evan Kovac, Robert E. Weiss, Wadih Arap

**Affiliations:** 1 Rutgers Cancer Institute, Newark, NJ USA; 2 Division of Urology, Department of Surgery, Rutgers New Jersey Medical School, Newark, NJ USA; 3 Division of Hematology/Oncology, Department of Medicine, New Jersey Medical School, Newark, NJ USA

## Introduction

In the realm of urologic oncology, the classification and terminology of prostate cancer stand as pillars guiding diagnosis, treatment, and patient communication. However, amidst the evolving landscape of cancer understanding, it’s imperative to reassess the language we employ to describe the disease. Gleason 6 prostate cancer, long regarded as the archetype of low-grade malignancy, deserves a nuanced and precise term that reflects its true nature and mitigates unnecessary fear. Enter PrNLMP: Prostatic Neoplasm of Low Malignant Potential.

## Revisiting Gleason 6

Gleason 6 prostate cancer has historically been viewed as a low-risk entity, often associated with indolent behavior and a favorable prognosis. This sentiment is underscored by studies indicating a minimal risk of metastasis to seminal vesicles or lymph nodes in Gleason 6 cases [1].

Such findings challenge the conventional understanding of prostate cancer as a uniformly aggressive disease and highlight the need for tailored approaches to management. According to the American Urological Association (AUA) and National Comprehensive Cancer Network (NCCN), active surveillance is the preferred treatment for low-risk disease [2, 3]and this strategy carries an excellent long-term survival profile.

## The Case for PrNLMP

By adopting the acronym PrNLMP, we acknowledge the distinct biological characteristics of Gleason 6 prostate cancer while reframing its classification in a manner that aligns with its clinical behavior. The term “neoplasm of low malignant potential” accurately captures the minimal propensity for aggressive behavior exhibited by Gleason 6 tumors without dismissing their potential for progression or harm.

The introduction of the term PrNLMP for Gleason 6 prostate cancer bears resemblance to the concept of PUNLMP (Papillary Urothelial Neoplasm of Low Malignant Potential) in bladder pathology. Just as PUNLMP distinguishes low-grade urothelial lesions with minimal malignant potential from more aggressive forms of bladder cancer, PrNLMP seeks to differentiate Gleason 6 prostate cancer as a neoplasm of low malignant potential deserving of tailored management strategies. Both terms reflect a paradigm shift towards precision medicine, emphasizing the importance of accurate characterization and risk stratification in guiding clinical decision-making. By drawing parallels to the successful implementation of PUNLMP in bladder pathology, the adoption of PrNLMP offers a compelling framework for redefining the classification and perception of Gleason 6 prostate cancer within the urologic community.

## Surveillance and Management

It’s important to emphasize that the adoption of PrNLMP does not diminish the need for vigilance and surveillance in patients with this diagnosis. While Gleason 6 prostate cancer may exhibit a relatively indolent course, the possibility of disease progression necessitates ongoing monitoring and informed decision-making regarding treatment options [4, 5]. Active surveillance remains a cornerstone of management for PrNLMP, offering a balance between the avoidance of overtreatment and the timely intervention in cases of disease evolution [2].

## Improving Diagnostic Accuracy

In the modern era of MRI-targeted fusion biopsy, our ability to accurately sample the prostate has markedly improved. This enhanced precision increases our confidence in ruling out the presence of higher-grade disease that may otherwise be missed on systematic biopsy. Consequently, the likelihood of misclassifying more aggressive cancer as Gleason 6 is significantly reduced, reinforcing the biological indolence of true Gleason 6 lesions [6].

Additionally, the incorporation of PSA dynamics and molecular biomarkers has further refined risk stratification. These tools assist clinicians in predicting tumor behavior and personalizing surveillance strategies, ensuring that patients with Gleason 6 disease receive appropriate monitoring without unnecessary intervention [7].

## Shaping Patient Perspectives

Beyond its implications for clinical practice, the adoption of PrNLMP holds significant implications for patient communication and empowerment. By reframing Gleason 6 prostate cancer as a neoplasm of low malignant potential, we can alleviate the undue anxiety and distress often associated with a “cancer” diagnosis. Patients may find solace in understanding that their condition is characterized by a low likelihood of aggressive behavior, thereby fostering a sense of optimism and control over their health journey.

## Challenges and Considerations

Of course, the transition to PrNLMP is not without its challenges. Skepticism and resistance may arise within the urologic and pathologic communities, stemming from concerns regarding terminology standardization and the potential for confusion among clinicians and patients. However, with careful education and dialogue, we can navigate these obstacles and foster widespread acceptance of PrNLMP as a clinically meaningful and patient-centric term.

## Resistance from the Pathology Community

While the adoption of PrNLMP holds promise for streamlining terminology and improving patient understanding, it may encounter resistance from some members of the pathology community. Pathologists play a pivotal role in the diagnosis and classification of prostate cancer, and any proposed changes to terminology are met with scrutiny and caution.

One potential source of resistance stems from the established conventions and familiarity associated with the Gleason grading system. Developed by Dr. Donald Gleason in the 1960s, the Gleason score has served as the gold standard for prostate cancer grading for decades [5]. As such, any deviation from this widely accepted system may be viewed as unnecessary or disruptive by pathologists accustomed to its use. More recently, Epstein and colleagues have proposed the concept of Gleason groups in which the Gleason 6 score corresponded to the Gleason 1 grade group [4].

Moreover, pathologists may express concerns regarding the potential for confusion and ambiguity resulting from the introduction of a new term like PrNLMP. Standardized terminology is essential for effective communication among healthcare providers and consistency in reporting and research. The introduction of PrNLMP alongside existing classifications such as Gleason scores and International Society of Urologic Pathology (ISUP) grades could lead to fragmentation and discordance in reporting practices, raising challenges for data interpretation and comparison.

Additionally, pathologists may question the scientific basis for reclassifying Gleason 6 prostate cancer as a neoplasm of low malignant potential. While studies have demonstrated the relatively indolent behavior of Gleason 6 tumors, some pathologists may argue that the term “cancer” accurately reflects the histological features and potential for disease progression observed in these cases [7]. Resistance to reclassification may stem from a desire to uphold rigorous diagnostic standards and avoid the perception of downgrading or minimizing the significance of certain lesions.

## Addressing Resistance

Addressing resistance from the pathology community will require thoughtful engagement, collaboration, and evidence-based discourse. Pathologists should be actively involved in discussions surrounding the adoption of PrNLMP, with opportunities for input and feedback throughout the decision-making process. Education and training initiatives can help familiarize pathologists with the rationale behind the proposed terminology change and provide guidance on its implementation in clinical practice.

Furthermore, efforts to validate and standardize the use of PrNLMP through multicenter studies and consensus-building initiatives can enhance its acceptance and utility within the pathology community. By demonstrating the clinical relevance and prognostic implications of PrNLMP in large-scale cohorts, researchers can bolster confidence in its use as a meaningful descriptor for Gleason 6 prostate cancer.

In navigating potential resistance from the pathology community, it’s essential to approach discussions surrounding the adoption of PrNLMP with sensitivity, collaboration, and a commitment to evidence-based practice. By engaging pathologists in dialogue, addressing concerns, and providing robust scientific justification, we can work towards consensus and acceptance of PrNLMP as a valuable addition to the lexicon of prostate cancer terminology.

## Legal and Liability Considerations

While we advocate for the adoption of PrNLMP as a more biologically accurate and patient-centered term, we recognize that such a shift introduces potential legal and liability challenges. Reclassifying Gleason 6 prostate cancer may raise concerns regarding informed consent, malpractice exposure, and the evolving definition of standard of care.

One potential concern is whether omitting the term “cancer” could later be (mis)construed as a failure to disclose a serious diagnosis. Physicians must ensure that patients understand that PrNLMP, though indolent, still requires monitoring and may progress in rare cases. Transparent discussions and precise documentation will be key to maintaining trust and avoiding legal misunderstandings.

At the same time, continuing to label these tumors as “cancer” risks overtreatment, with associated complications, costs, and emotional burden—all factors that can give rise to malpractice claims. In this context, PrNLMP may offer a legally defensible alternative that better aligns treatment decisions with actual tumor behavior.

Ultimately, legal frameworks and medical standards evolve *in tandem*. To support this terminological shift, professional societies and regulatory bodies must develop clear guidance that ensures consistency across clinical care, documentation, and patient communication. Raising awareness of these issues is a necessary step toward broader acceptance of PrNLMP in both clinical and legal contexts.

## Conclusion

In reimagining Gleason 6 prostate cancer as PrNLMP, we take a bold step towards precision and progress in the field of urologic oncology. By embracing a term that accurately reflects the clinical behavior of these tumors, we empower both clinicians and patients to approach diagnosis and management with clarity, confidence, and compassion. As we continue to refine our understanding of prostate cancer, let us remain committed to language that honors the complexities of the disease while prioritizing the well-being and dignity of those affected.

## Data Availability

No datasets were generated or analysed during the current study.
